# “More than just a medical student”: a mixed methods exploration of a structured volunteering programme for undergraduate medical students

**DOI:** 10.1186/s12909-021-03037-4

**Published:** 2022-01-03

**Authors:** Kerry Badger, Rory Morrice, Olivia Buckeldee, Natalia Cotton, Dilshani Hunukumbure, Oliver Mitchell, Ameer Mustafa, Ebun Oluwole, Juhee Pahuja, Daniel Davies, Mary J. Morrell, Sue Smith, Kathleen Leedham-Green

**Affiliations:** 1 Imperial College School of Medicine, Imperial College London, London, UK; 2 Chelsea and Westminster Hospital NHS Foundation Trust, London, UK; 3 The Hillingdon Hospitals NHS Foundation Trust, London, UK; 4 Imperial College Healthcare NHS Trust, London, UK; 5 National Heart and Lung Institute, Faculty of Medicine, Imperial College London, London, UK; 6 Medical Education Research Unit, Imperial College London, London, UK

**Keywords:** Volunteer, Service learning, Transformative learning

## Abstract

**Background:**

As a result of the COVID-19 pandemic Imperial College School of Medicine developed a structured volunteering programme involving 398 medical students, across eight teaching hospitals. This case study aims to explore the relationship between the processes, context, participant experiences and impacts of the programme so that lessons can be learned for future emergencies and service-learning programmes.

**Methods:**

Using an illuminative approach to evaluation we invited all volunteers and supervisors to complete a mixed-methods survey. This explored differences in experience across demographics and contextual factors, correlations between aspects of induction, supervision and overall experience, and reviewed the impacts of the programme. Quantitative responses were statistically analysed and qualitative reflections were thematically coded to triangulate and explain quantitative findings. Follow up interviews were carried out to check back findings and co-create conclusions.

**Results:**

We received responses from 61 students and 17 supervisors. Student participants described predominantly altruistic motivations and transformational changes to their professional identity driven by feeling included, having responsibility, and engaging in authentic workplace-based learning afforded by freedom from the assessed curriculum. They reported new perspectives on their future professional role within the multidisciplinary team and the value of workplace-based learning. They reported increases in wellbeing and self-esteem related to feeling included and valued, and positively contributing to service provision at a time of need. Significantly higher overall satisfaction was associated with a personalised induction, active supervision, earlier stage of training, and male gender. Gender-related differences were not explained through our data but have been reported elsewhere and warrant further study. The duration, intensity and type of role that volunteers performed was similar across demographics and did not appear to modulate their overall experience.

**Conclusions:**

Whilst acknowledging the uniqueness of emergency volunteering and the survey response rate of 15% of volunteers, we suggest the features of a successful service-learning programme include: a learner-centred induction, regular contact with engaged and appreciative supervisors, and roles where students feel valued. Programmes in similar settings may find that service learning is most impactful earlier in medical students’ training and that students with altruistic motivations and meaningful work may flourish without formal outcomes and assessments.

**Supplementary Information:**

The online version contains supplementary material available at 10.1186/s12909-021-03037-4.

## Introduction

COVID-19 has placed unprecedented pressure on healthcare systems across the world. During the first wave of the pandemic, UK medical students, although encouraged to focus on their education, were offered the opportunity to support the National Health Service (NHS) [1]. At Imperial College London School of Medicine (ICSM) a volunteering programme was rapidly developed across eight teaching hospitals and the community to enable medical students in all years to support the NHS workforce within a structured environment. The primary aim of this volunteering programme was service provision. These students experienced a shift in role from medical student to volunteer, or service provider, and this created unanticipated transformative educational value for many volunteers. This case study aims to illuminate the experiences of volunteers and the contexts and mechanisms of learning, including these unanticipated outcomes, drawing lessons for future emergencies as well as for curriculum improvements.

Volunteer service-learning activities are commonplace in medical education programmes worldwide including first responder programmes, student-led clinics and public health outreach programmes [2–4]. While involvement with service learning and service provision have been shown to increase students’ self-rated knowledge and skills [5] and increased understanding of health equity and the social determinants of health [6], little is known about the processes that underpin this learning. There is longstanding critical debate about using medical students as volunteers, or unpaid workers, and how to balance the needs of the service with the needs of the learner [7–9]. Furco describes a spectrum of service programmes with internships at one end of the spectrum (where the focus is on the learner) and volunteerism at the other (where the focus is on the service) [9]. There remains a gap in the literature for empirical evidence on what educational value is created on the service-provision end of that spectrum, and the impacts of context and process on student experience.

The Imperial College School of Medicine (ICSM) volunteering programme involved 398 students from all years, distributed across eight teaching hospitals and the community. This is an exploration of the hospital-based component.

## Methods

### Aim

We aimed to explore how a shift in role identify from learner to volunteer impacted on medical students’ hospital-based experience and learning. Our secondary aim was to explore differences in experience across demographics and contexts. Who did the programme work for, in which contexts and why?

### Methodology and theoretical framing

Our aim was to explore any learning that occurred, including unintended outcomes, rather than learning gain against predefined objectives, and to document volunteer experience, including both supervisor and volunteer perspectives, so that processual lessons could be learned. By looking at contexts and processes as well as impacts and outcomes, it becomes possible to theorise about the mechanisms of learning including modulating factors. We wanted to describe the programme from within, including the perspectives of both volunteers and supervisors who were involved as co-creators of the evaluation. This illuminative approach to evaluation [10] allows for progressive focussing in response to unpredicted phenomena, so theoretical framing is developed in response to the data. Our analysis was ultimately framed by theories of service learning, motivation and role identity.

### Context

This study was conducted across eight teaching hospitals in North West London following a structured volunteering programme for medical students organised by senior faculty of ICSM and the Trust Hospitals in North West Thames, during the first wave of the COVD-19 pandemic in March – July 2020.

ICSM runs a 6-year integrated MBBS course with early clinical exposure from its first year. As a result of COVID-19, medical student clinical placements were suspended on 13th March 2020. Students from all year groups were offered the opportunity to volunteer, although scheduled teaching continued. Following extensive planning, including individual risk assessments, the students started their first shifts on 31st March 2020. Volunteers each had a named Clinical Supervisor and each site had an allocated Clinical Teaching Fellow who was supported by the Director of Clinical Studies to oversee the programme. ICSM also provided named Tutors for each hospital, who were available for additional support. The programme included an offer of free accommodation so that students with long commutes or with vulnerable household members could be included. The programme was not integrated to the curriculum and as such there was no formalised curriculum, learning objectives or assessments. In the United Kingdom, guidance relating to use of medical student volunteers was released from three national bodies, the General Medical Council (GMC) [11], the British Medical Association (BMA) [12] and the Medical Schools Council (MSC) [13]. These documents set out themes of appropriate supervision to work within competency, adequate PPE provision and students’ ability to withdraw from their roles at any time. GMC guidance states that ‘students must be supervised to be safe, act within their competence and must not undertake duties of a doctor. The safety of patients and students must be the first priority’ [11]. While some medical schools chose to formally employ medical students as healthcare assistants, ICSM adopted a volunteering approach [1]. ICSM worked closely with the BMA to ensure student and patient safety remained the priority. Additional file 1 outlines the timeline the volunteering programme, as well as the evolving COVID-19 pandemic (Additional file 1).

### Participants

All medical students involved in ICSM’s structured hospital-based volunteering programme, their clinical supervisors and associated support staff were invited to participate in this evaluation. The roles of student volunteers varied according to volunteer preference, local needs and the clinical competency of the students across the 6 years of their medical training. 35% of respondents took on clinical roles within the MDT e.g. phlebotomy; 23% performed medical roles e.g. ward assistant to a doctor; 26% had non-clinical roles e.g. PPE distribution or administration and 16% had a mixed role.

### Data generation

KB and KLG developed the evaluation strategy in collaboration with students, clinical teaching fellows, faculty and administrators. We chose a survey so that we could capture all volunteers’ and supervisors’ experiences immediately after the programme finished, and a mixed methods design so that we could measure, explore and explain differences in experience. Its scope was informed by discussions across the research team, seven of whom had spent time supervising and debriefing volunteers, and with two volunteer medical students. Our research questions were further informed by the administrative and academic leads for the programme who were interested in what value had been created, not just for learners, but for patients, services and the Medical School, plus the impacts of processual factors so that lessons could be learned for the future.

We drafted the survey in Qualtrics and tested it across the research team and with two medical students who commented on its scope, acceptability, length, clarity, and usability. This resulted in a reduction in the number of free-text boxes, a simpler star grading for the Likert scales, and refinements to wording. We designed a similar survey for clinical supervisors and support staff, reframing the questions from their perspective, which was checked and refined by the scheme administrator. The surveys are shown in an additional file (Additional file 2). We obtained ethical approval and distributed the survey to all volunteers, supervisors and support staff by email the week the volunteering programme finished, with follow-up invitations and shoutouts over the following six weeks. Following survey data analysis EO and RM conducted a member-checking phase with two volunteers who self-selected to participate in a semi-structured one to one interview to check back findings and involve stakeholders in co-creating our conclusions.

### Data analysis

**Fig. 1:**
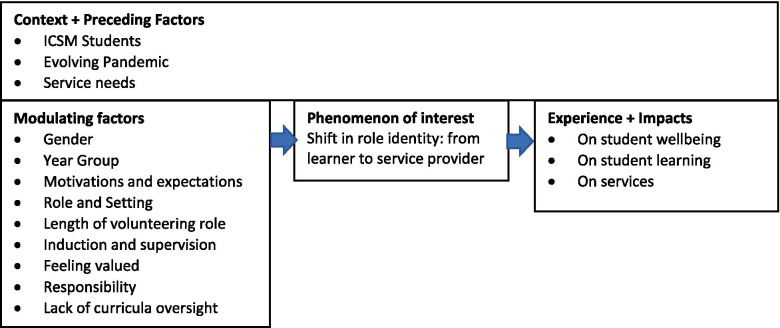
Coding Framework

We coded qualitative responses at a team coding day assisted by Zoom videoconferencing and Dedoose, a cloud-based qualitative analysis program, which allows multiple users to code concurrently. We adopted a consensual research approach where each statement was discussed and coded with its paraphrased meaning and content codes were iteratively refined, merged where appropriate, and sorted into themes. The themes were categorised into an overarching framework (Fig. 1) through a process of iterative discussion. Comparisons were made between positive and negative experiences to explore the impacts of modulating factors, and across demographics to explore the influence of preceding factors. The coding was audited and refined by AM and the categories, themes and subthemes refined by KB and KLG. Contextual factors were supplemented with data from the administrative and academic leads for the programme, as well as publicly available data relating to the evolving pandemic.

Statistical analysis was facilitated by ‘R for Statistical Computing, Version 4.0.3’ software. We investigated whether participants’ overall rating of the experience varied according to demographic groups and potential modulating factors to their experience (Table 1) using Mann-Whitney U-tests. We combined free text coding with drop down responses to create categorical groups according to stated reasons for volunteering (to help/to learn/to stay busy/mixed), start date (early i.e. March 2020/middle i.e. April 2020/late i.e. May 2020) and duration of volunteering (short i.e. < 4 weeks /long i.e. > 4 weeks), role (non-clinical / clinical – MDT / clinical - Assistant Doctor / mixed), stage in training (years 1 and 2/ years 3–6). We investigated how specific aspects of participants’ induction and supervision affected their overall rating by testing the Spearman’s rank correlation between Likert scores and their overall rating. We corrected for multiple comparisons using the Holm correction. Where significant associations were found, we examined qualitative comments to explain causality.

**Table 1:** Characteristics of student participants

Characteristics	N (%)
Gender	
Male	34 (56)
Female	27 (44)
Identify as minority ethnic	
Yes	32 (52)
No	26 (43)
Prefer not to say	3 (5)
Identify as from a social background where few people go to university	
Yes	14 (23)
No	45 (74)
Prefer not to say	2 (3)
Year Group of Study	
1	2 (4)
2	16 (26)
3	11 (18)
4	8 (13)
5	21 (34)
6	3 (5)

## Results

For ease of reading, we present theme-specific interpretations alongside our results and overall interpretation is presented within the discussion. We report verbatim quotes in italics, identifying participants by their role (Volunteer (V); supervisor (S)) and a numerical identifier. Contractions and clarifying insertions are indicated with ellipses and square brackets.

We received 61 survey responses from 398 volunteers (Table 1) and 17 responses from supervisors. This low response rate means that demographics are unlikely to be representative of the whole cohort. We report statistical analysis of quantitative responses for the 61 volunteer responses below (Tables 2 and 3). This analysis assumes that participants within each demographic category are representative of that category. These findings must therefore be interpreted with caution and are used here to support and triangulate qualitative insights.

**Table 2:** Variance in volunteer rating according to volunteer demographic

Variable	N (no)	N (yes)	N (Missing/Prefer not to say)	Mean Volunteer Rating “no” (SD)	Mean Volunteer Rating “yes” (SD)	*P*-value	P-value (Holm-corrected)
Male	27	34	0	4.3 (0.7)	4.8 (0.4)	0.006	0.045
Ethnic minority background	32	26	3	4.5 (0.6)	4.7 (0.5)	0.114	0.572
Background where few people go to university	45	14	2	4.6 (0.6)	4.7 (0.5)	0.485	0.78
Motivation purely to help	18	43	0	4.5 (0.6)	4.6 (0.6)	0.268	0.78
Started early (i.e. start date in March 2020)	31	29	1	4.8 (0.4)	4.4 (0.7)	0.024	0.146
Volunteer duration 4 weeks or less	19	36	6	4.4 (0.7)	4.6 (0.5)	0.218	0.78
Years 1 or 2 student	43	18	0	4.4 (0.6)	4.9 (0.2)	0.002	0.013
Clinical role	16	45	0	4.7 (0.6)	4.5 (0.6)	0.195	0.78

**Table 3:** Comparison of individual aspects of participants’ experiences and their overall rating of the experience

Item	Correlation with volunteer rating (r)	*P*-value	*P*-value (Holm corrected)
Induction appropriately prepared for role	0.17	0.2	0.399
Induction increased my confidence	0.31	0.02	0.107
Induction opportunity to discuss role	0.41	0.002	0.015
Induction opportunity to discuss questions	0.38	0.004	0.029
Induction adequate guidance	0.32	0.018	0.107
Induction adequate PPE training	0.3	0.024	0.107
Supervision felt adequate	0.17	0.202	0.399
Regular meetings with supervisor	0.41	0.001	0.012
Good rapport with supervisor	0.21	0.108	0.324
Role support from supervisor	0.48	< 0.001	0.001
Personal support from supervisor	0.34	0.009	0.062

Participant characteristics are listed in Table 1, comparison of experience across demographic groups in Table 2, and correlation between potential modulating factors and overall experience in Table 3.

Table 2 shows results of Mann-Whitney U-tests comparing whether the overall rating of the volunteering experience (volunteer rating) varied according to demographic features or other features of the experience. *P*-values are reported uncorrected and corrected for multiple comparisons using the Holm correction. Participants with missing data or who indicated they preferred not to answer were omitted from comparisons.

Table 3 shows Spearman’s rank correlations between individual aspects of participants’ experiences and their overall rating of the experience. We report uncorrected p-values and *p*-values corrected for multiple comparisons using the Holm correction.

### Modulating factors

#### Gender

Male students reported significantly more positive overall ratings of experience (Table 2). Qualitative and quantitative data did not illuminate this finding. Both genders were allocated to similar roles and there were no reports of direct discrimination. However further research is needed to exclude unconscious biases, for example in expressions of gratitude, and to explore and explain this further. Interestingly, this finding is consistent with previous findings of men experiencing greater increases in wellbeing during emergency volunteering activities [14].

## Year group

Students in years 1 and 2 reported significantly more positive overall ratings of the experience when compared to students in years 3–6 (Table 2). Qualitative comments reveal a notable difference between earlier years and more senior students’ fears. More senior students worried about working outside their competency: *“I was obviously worried about making the lives of doctors harder if I was deemed incompetent”* V10 (Year 5 student)*.* While, those in years 1 and 2 found they were more useful than anticipated: “*I was much more useful than expected and with the appropriate support from ward staff and the clinical fellows I was able to make a genuine difference to a lot of people*” V48 (Year 2 Student).

These comparatively lower expectations of years 1/2 students may have led to the more positive perception of their experience. This further validates recommendations from the literature that service learning should be incorporated into years 1 and 2 [15, 16].

### Motivations and expectations

Overwhelmingly, students described altruistic motivations for volunteering, with 98% citing a desire to help: *“From the very beginning of the pandemic I felt the urge to help in any way”* V47*.* Some felt it was their duty to utilise their clinical skills: “*As a medical student, I felt it was my duty to help the NHS and Imperial Trusts as much as I can in the face of a pandemic*” V3. As a result, many hoped to be able to provide a valuable contribution “*I hoped to do jobs that the NHS didn’t have the resources to do without volunteers*” V49. 28% of respondents also co-expressed more self-directed motivations for volunteering such as a desire to learn or to alleviate boredom: “*I had nothing else to do*” V18. There was no statistically significant difference in overall volunteer rating for those expressing a mixed motivation for volunteering when compared with those whose motivation was purely altruistic. The uniformity of volunteers’ altruistic motivations for participation may relate to this being a voluntary programme and the unprecedented circumstances of the pandemic.

Participants also discussed how their positive experience relate to their motivations:

*The experiences gained were invaluable and unlike anything learnt on placement where you are not there out of freewill*” V13. Overall 95% of students gave an overall volunteer rating of 4 or 5 out of 5 stars and similarly 98% cited altruistic motivations.

Links between motivation, satisfaction and learning are well documented. Self-determination theory [17] suggests that intrinsic and internalised extrinsic motivation is dependent on the satisfaction of three basic psychological needs; need for autonomy, competence and relatedness. Through enabling volunteers to choose their roles, having a genuine need for volunteer assistance and the sense of belonging experienced by students, as a result of the reciprocal benefits for both volunteer and service, there was fulfilment of these needs and this may explain the success of the programme. Therefore, the high satisfaction expressed by volunteers within this programme may not translate to service-learning programmes where student’s motivations are more egoistic.

### Timing and length of volunteering role

The number of volunteering shifts per week that each participant completed varied and the median length of volunteering was 4 weeks, with a range of 1–10 weeks. We did not find any differences in satisfaction based on either the length of time spent volunteering, whether they started their role early (i.e. during March 2020) or the number of shifts they did per week.

### Induction and supervision

All volunteers completed a mandatory one-day online induction delivered by ICSM, followed by an onsite face-to-face induction at each hospital. Most volunteers felt the induction appropriately prepared them for their roles, with 79% rating this statement 4 or 5 out of 5 stars. The 12% of participants who rated the same statement below 3 stars, cited either practical issues being inducted into the wrong areas, or difficulty accessing equipment. Statistically significant positive correlations were found between volunteers being able to discuss their role and ask questions during local induction and participants overall volunteering rating (out of 5 stars) (Table 3). Role identity was a theme here: “*more direct briefing to the doctors of our role would have helped as often we weren’t being used and were treated like we were on placement*” V19. Additionally, some volunteers felt the induction could have done more to prepare them for the emotional aspect of treating Covid-19 patients. “*I wish we had been prepared a bit more for the reality of COVID and the deaths. Just a lecture in the introduction to acknowledge the difficulties of the treatment and the potential emotional side of it*” V9.

Positive correlations were demonstrated between having regular meetings with their Clinical Supervisor and having role support from their supervisor. These associations were maintained after correction for multiple comparisons (Table 3). Relationships with clinical supervisors appeared to be modulated by this shift in role: volunteers described being “*treated me like a peer*” V31 and “*[My supervisors] felt like friends by the end of volunteering”* V57. Others felt they did not require structured supervision and noted a feeling of belonging within teams as the reasoning for this: “*I didn’t feel like I needed to have specific support as the junior team I was in was incredibly supportive already*” V26. Conversely, sub-optimal supervision impacted negatively on one volunteer’s experience: “*There wasn’t enough supervision, and I rarely had any for clinical tasks I was asked to do. I didn’t agree to do anything I hadn’t been signed off for on previous placements, but there was rarely the option of me doing a procedure (e.g. cannulating) with supervision*” V4.

Therefore, our data aligns with established findings about the importance of appropriate induction and supervision, particularly at times of transition [18]. We reflect on the success of these processes within the volunteer programme and further research may be warranted to build on our finding of positive experiences of informal supervision within clinical teams.

### Role responsibility

The role the student performed did not have a significant effect on students’ overall volunteer rating (Table 2). Qualitative analysis highlighted that some students in less pressurised environments remained in a learner role: “*it felt more like a clinical placement that I would have in third year, as the staff were not as busy as I’d expected and had time to teach us*” V41. Others in busier pressurised environments, described how challenging experiences were mitigated by feeling involved, useful or helpful “*They were very often thanking me for being there and helping which felt good. I also think doing some of the easier tasks meant that it relieved pressure on the senior members of the team so they could take time to do the more complex tasks*”. After the first wave of the pandemic subsided ‘No longer feeling useful’ was cited as a reason for leaving the scheme by 44% of respondents, suggesting this as an important factor motivating participation.

As volunteers, participants described a higher level of entrustment than as a student, citing staff workload as a factor in this:

*everyone had their metaphorical plates so full that they didn’t have any more room to watch over me largely, and thus any mistakes I made would truly be my own -* V11

The entrustment felt appears to have empowered them and given them a sense of belonging:

*[As a medical student] we don't have any responsibility and so we never feel as involved with the team … you often hide behind the doctors whereas when volunteering I felt able to make these small clinical decisions -* V23.

*This is the first time I've been in a hospital and felt properly valued. Everyone in the team knew my name, acknowledged my presence and made me feel welcome. I felt part of the team. -* V26.

They linked feeling useful to the quality of their learning experience, and to team inclusion:

*I think the entire difference is down to the fact that in volunteering it is a mutually beneficial arrangement whereas on placement doctors can often view you as an added burden to their already high workload* - V19.

Others emphasised the relationship between the value they attributed to their role and its perceived relationship to their expectations, with many expressing dissatisfaction if they did not feel they were directly contributing to the pandemic response “*I couldn’t see how that was a direct COVID related job. Therefore, the role didn’t feel meaningful*” V49.

Valuable contribution and subsequent entrustment within the community of practice developed through service-based learning is cited as an important motivator [18] and our results suggest that the actual role performed by volunteers is less important to outcomes than this perceived responsibility of the role. Such accounts appear to validate the theory that active participation in the workplace is reliant on both individual willingness and to how the workplace affords participation [19] as well as the importance of belonging and reciprocity [20] in value creation.

### The move from formal to informal learning

Both volunteers and service staff discussed that having no formal curriculum or assessment requirement enabled better integration within clinical teams and a more patient-centred approach to their role. They cited increased time spent on the ward and students’ motivations shifting away from assessment outcomes and towards ‘being helpful’ as an important factor:

*Being based on a single ward with no teaching/ other educational commitments meant they got to know their patients in much greater depth -* S3*.*

*There were fewer restrictions when learning as a volunteer. This allowed us to get involved more and become increasingly comfortable in our roles ... As a student there's more focus on teaching and testing your understanding and less taking initiative in patient care -* V61.

When we checked back our findings, one volunteer further noted:

*I got to know the patients extremely well, so I wanted to learn how to change an adult nappy without having an extra person. This was not my primary aim as a student.-* V6.

Frequently educational literature emphasises the importance of curriculum alignment and appropriate assessment [16]. This thinking contrasts with the success of this out of curriculum volunteering programme. This phenomenon is therefore better explained using self-determination theory [17]. Whilst we acknowledge the impact of the unique context of the programme on the self-determination of students and therefore that these finding may not be transferable to programmes in contexts outside of emergency response, we focus on harnessing self-determination in students as a method to improve educational outcomes.

### Impacts

#### Volunteer and staff wellbeing

Many reported positive impacts on their wellbeing, linking this to peer interaction, having a valued role, as well as a sense of camaraderie: *“My well-being improved greatly from volunteering as I was able to interact with a wide variety of people and make friends with fellow volunteers”* V41, “*It provided me a great sense of purpose*” V47.

Exposure to the distressing clinical context proved difficult for some: “*When you care for a patient yourself… when they die or get worse or their family demand answers, it really really hurts*” V6. Distress was mitigated by supportive supervision and debrief: *“I think the biggest impact was that I saw a lot of deaths, and patients’ families grieving. I found this really challenging, but my supervisors were very supportive, as were the people on my team”*V37.

Several staff also reported improved wellbeing related to the scheme: “*I actually think that having student volunteers boosted morale as they were so enthusiastic to have around that everyone really appreciated more hands!”* S12.

This positive impact on volunteer and staff wellbeing, in spite of the challenging emotional situation, supports findings from longitudinal studies that volunteering contributes to psychological well-being, which may be attributed to experiencing reciprocal benefits and the social nature of volunteering [21, 22].

#### Volunteer learning

The volunteering programme appeared to have a profound impact on volunteer learning and professional identity formation as well as cognitive-emotional development:

*Volunteering during the pandemic showed me what it means to be a doctor more than any clinical placement* - V13

*I can't emphasize how beneficial I feel this has been for the Y6 students - I have really seen them grow and they are more than ready for FY1 now* - S13

In addition to the development of practical skills and knowledge, volunteers consistently expressed transformational perceptual shifts. The strongest transformative themes related to:

1. Reframing self as competent and effective:
  *My confidence in my own abilities has increased greatly* - V12;
2. Reframing professional role as within a team:
  *I think this experience has taught me how to appreciate and respect all members of a team* - V12,
  *They developed huge appreciation of the role of the entire hospital workforce in keeping a hospital going* - S7; and
3. Reframing their approach to effective clinical learning:
  *It is just better on every level – by actively doing, you learn more, and by being a help to the team they value you and have more interest in supporting and teaching you. - V19*
  *They learned that such active involvement can be rewarding and fulfilling as well as extremely beneficial to their learning - S12.*

Transformative learning within service-based roles has been repeatedly demonstrated within medical education [23]. Context and environment are integral to facilitating this, with safe learning environments that promote self-reflection and encourage the learner to reframe their thinking viewed as the most effective [24]. Therefore, perhaps the transformative learning that occurred may be partially attributed to the volunteering programme creating an appropriate environment for this to occur.

#### Service contribution

The scheme administrators reported that requests for student volunteer assistance outweighed the supply of volunteers at the peak of the pandemic, related to extra-ordinary service demands compounded by staff absences due to illness or isolation. Student volunteers were therefore viewed as integral to maintaining services: ‘*The hospital would have struggled without their support!*’ S7. Many supervising staff stated they could have benefitted from more volunteers and sooner: ‘*We needed the students help much earlier’* S7 and ‘*more students will be helpful if a second wave happens’* S6.

Despite supervisors fears of the extra workload of supervising student volunteers, some found this was not as burdensome as anticipated and that the negatives were far outweighed:

*[students were] less demanding and [had more] well-balanced student expectations than the usual you get in clinical attachments* - S15.

Supervisors noted the impact of the change in role on engagement:

*Usually with our medical student attachments the students need quite a lot of encouragement to attend the wards/ spend prolonged time in clinical environments but in their volunteer capacity we found that the students integrated well within teams and found a clear role for themselves. This was great to see!* - S11.

During the check-back interviews one clarified how this commitment to service provision led to a sense of community and positive professional identity formation:

*Seriously. I didn't know any of the other students I worked with before volunteering, but all of them 100% committed and will make inspirational doctors in the future* - V6.

This valuable contribution of students enabled them to feel a sense of belonging within communities of practice and this in turn lead to transformative learning effects, in particular a reframed view of effective clinical learning was noted by supervisors [16, 20]. This reframing perhaps enabled increased workplace acceptance of volunteers [20] and subsequently volunteers experienced a reciprocity of benefits within service-based learning in a way which had not been possible as a medical student.

## Discussion

### Summary of findings

**Fig. 2:**
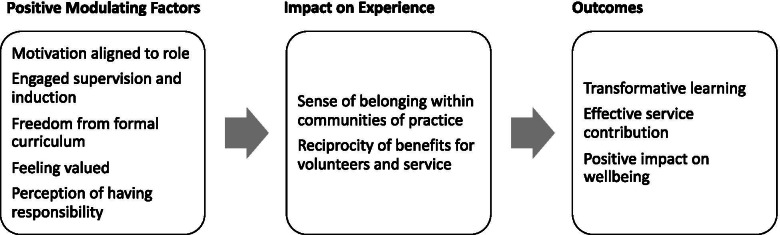
Summary of findings

Our data suggested that gender and year group are important modulating factors in student volunteer satisfaction, however the role performed (i.e. clinical vs non-clinical) and length of volunteering (1–10 weeks) did not significantly impact outcomes. Volunteers described the impact of altruistic motivations on outcomes and moving away from their relatively protected and powerless role as learner towards a role where they felt both useful and responsible. This created transformational shifts in their attitudes to workplace-based learning, their approaches to working within a multidisciplinary team, as well as positive impacts on wellbeing and professional identity formation. Hospital staff described reciprocal benefits which were both practical and emotional, and exceeded the effort required for supervision. Figure 2 provides a summary of these findings.

### Discussion in relation to the literature

We have presented some of our discussion in line with our results where this was relevant to an individual theme. Our overall findings resonate strongly with Stewart and Wubenna’s analysis: “A value of engagement from service-learning is that medical students may experience transformative learning vis-à-vis cognitive-emotional development. As individuals critically reflect on their assumptions and beliefs, they can change their frames of reference, which can result in a fundamental change in the basic premises of thoughts, feelings and actions” P154 [16]. They conclude that emotions are inseparable from cognition, and that a sense of belonging and pride in achievement are drivers of engagement in authentic workplace-based practices and therefore learning.

Contrary to Stewart and Wubenna’s conclusion that service learning should align with defined learning goals which are assessed [16], we have found that both volunteers and staff benefitted from freedom from the formal curriculum. Our findings therefore point to a further (or perhaps alternative) condition of success: that there is a genuine reciprocity between volunteers and those that they seek to help: learners must feel motivated, useful and responsible, and the services accommodating them must have a genuine need for, and appreciation of, volunteers. In this instance where motivations were predominantly altruistic, we suggest that ‘being needed’, ‘feeling valued’ and ‘making a difference’ are alternative drivers of success, but where volunteers have primarily self-directed motivations for example learning, then agreeing and achieving those goals within their placement seems appropriate. This in turn may be related to context, the altruistic motivations of the self-selected participants and the extra-ordinary circumstances of the pandemic, and perhaps not replicable in a normal placement.

### Implications and applications for practice

We have organised our implications for practice into the four stages of Stewart and Wubenna’s (2014) framework for incorporating service learning into a medical curriculum (Table 4). These findings have relevance for similar emergency responses, and additionally implications for service learning within a wider curriculum.

**Table 4:** Insights and applications for practice

	Planning & preparation	Action	Reflection & demonstration	Assessment & celebration
**Insights from experience of a structured volunteering programme during the first wave of the COVID-19 pandemic**	- Emphasis on consultation of guidance from regulatory bodies for emergency response programmes - Emphasis on consideration of stage of training as years 1 and 2 benefit from service learning - Emphasis on a shift of focus away from formal curriculum oversight and towards ensuring roles in which students can make valuable contribution to maximise reciprocal benefits for services and students	Match roles to motivations	Emphasis on engaged supervision to support reflection	Emphasis on celebration

Table 4 shows insights from our structured volunteering programme during the first wave of the COVID-19 pandemic using the framework developed by Stewart and Wubenna’s (2014) for infusing learning into medical education.

During the ‘planning and preparation’ stage our experiences suggest it is essential to consult widely with all stake holders, to focus on appropriate guidance from regulatory bodies, carry out appropriate risk assessments and to assess the needs and preferences of both students and service providers. This is key to ensure safety but also acts to engage and elicit buy-in. During the ‘action stage’ we suggest it is important to identify motivations and expectations, so that students who are motivated by the need to help are paired with services that genuinely need them; and that students that are motivated by the need to learn have their learning goals agreed and appropriately assessed. From a service perspective, it is important to pair students’ capabilities and competencies with appropriate levels of responsibility. Our volunteer programme benefited from a dedicated team of support staff at ICSM, who managed the students’ needs and worked closely with the hospital sites. In the ‘reflection and demonstration’ stage we have identified appropriate engaged supervision with opportunities to debrief and reflect, and appropriate role support as integral to success. Our research suggests that students benefit from having a regular named supervisor, however supervision may be devolved to the wider team where it is clear that this is working effectively. In the final section of the framework, ‘Assessment and celebration’, we again reflect on the successes of this programme which had no formalised assessment. Whilst recognising that “assessment drives learning” we turn our focus towards the powerful motivators for student learning of feeling valued and responsibility that can be effectively cultivated within a service-learning role. Therefore, we identify with the importance of celebration and recognition as feeling valued was a key theme within our results.

### Strengths, limitations and implications for further research

Our study was sampled across year groups, roles and across hospital sites and the community. We had a balance of characteristics of student volunteers with representative proportions of different demographic groups included (Table 1). We recognise that this was a qualitative study carried out in one context and so our findings may not be transferable, in particular we recognise that the likely significant impact of the context within the COVID-19 pandemic. Additionally, student volunteers participating in this study had self-selected to undertake the volunteering experience, as well as to complete the survey and interviews. We have no way of comparing this group to those who did not participate. Further the response rate of 15% from student volunteers leaves our findings exposed to confounding and biases, especially resulting from attracting responses from volunteers who may been motivated to report experiences based in strongly negative and positive poles of the spectrum.

Our focus on role identity aligns with the focus of much previous research in the field of service learning and with the observational comments of supervisors and volunteers within the dataset. The focus of the impact of role identity to analyse the impacts from the volunteering programme has emphasised certain aspects of the data collected. Perhaps if we had utilised a different focus, such as the impact of motivation and expectations, different aspects of the data may have come to the foreground.

Further research could focus on faculty viewpoints on how to integrate these findings into the formal medical school curriculum. This is important as widespread agreement needs to be reached to produce change in curricula.

## Conclusion

While acknowledging the uniqueness of the situation presented by COVID-19 we suggest the features of a successful service-learning programme include: a learner-centred induction, engaged and appreciative supervisors, and the entrustment of students with meaningful work with reciprocal benefits to services. Our data suggests that gender and year group are important modulating factors in student volunteer satisfaction, however the role performed (i.e. clinical vs non-clinical) or length of volunteering (1–10 weeks) did not significantly impact outcomes. Programmes in similar settings may find that 1) volunteering is best appreciated in years 1 and 2, 2) students with altruistic motivations and meaningful work may flourish without formal outcomes and assessments, and 3) that female volunteers may experiences service learning differently to men. We are planning a follow-up study to confirm, explore and explain these differences. Further research is also warranted to outline with stakeholders how best to utilise the lessons learned from this programme in the formal curriculum, and to analyse the medium- and long-term impacts of the programme on participants.

## Supplementary Information

**Additional file 1. **Timeline in relation to contextual factors.

**Additional file 2. **Student and Supervisors, teaching fellows and clinical support surveys.

## Data Availability

Due to privacy concerns the full data set is not publicly available. A redacted version of the survey is available upon reasonable request.

## Abbreviations

BMA: British Medical Association
GMC: General Medical Council
ICSM: Imperial College School of Medicine
MSC: Medical Schools Council
NHS: National Health Service
