# Supplementary figures and images for: “More than just a medical student”: a mixed methods exploration of a structured volunteering programme for undergraduate medical students
Source: BMC Med Educ. 2022 Jan 3;22:1. doi: 10.1186/s12909-021-03037-4 (PMC8721190; doi:10.1186/s12909-021-03037-4)

## Additional File 1 – Timeline in relation to contextual factors

##
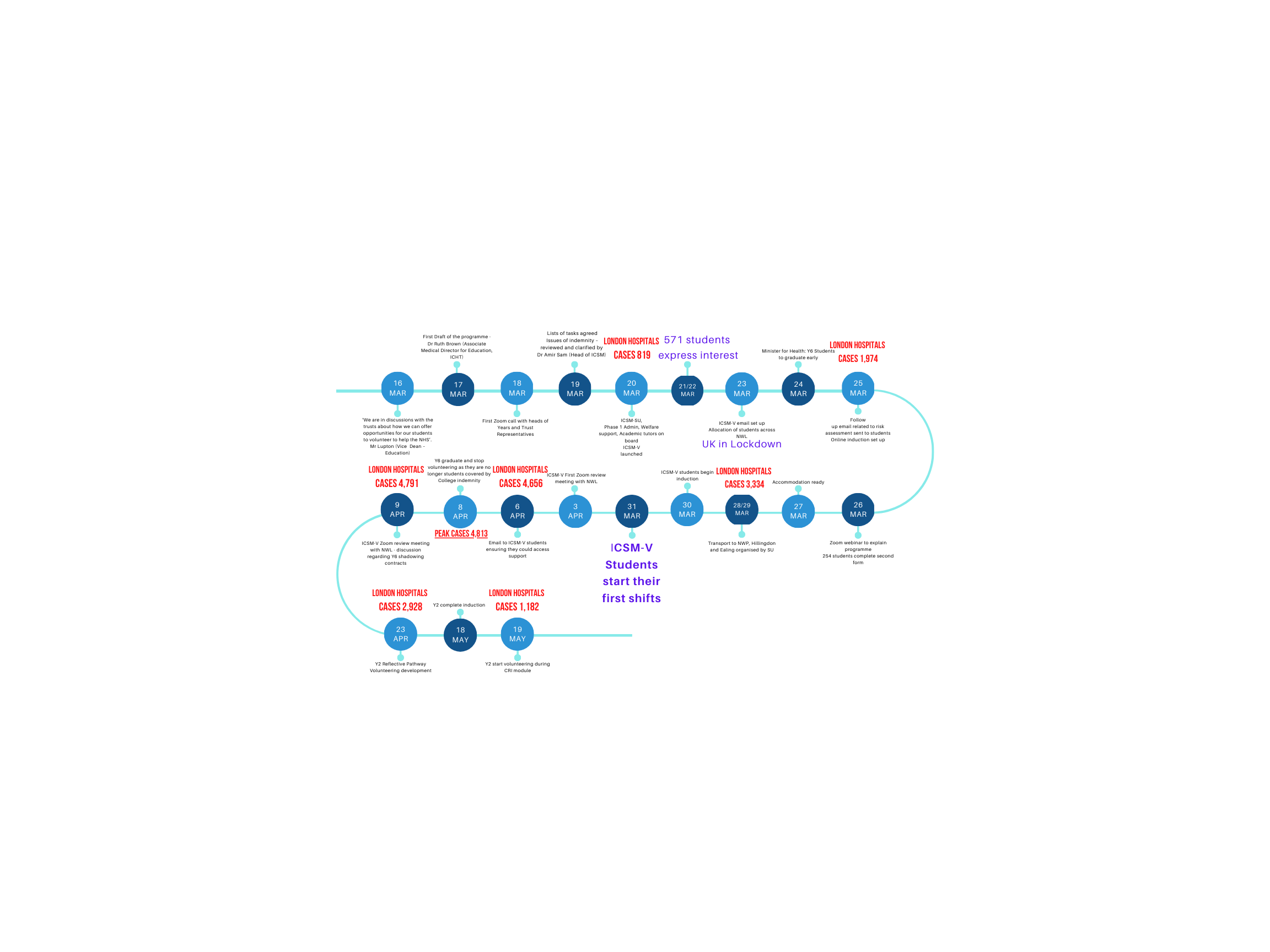

Supplement: Supplementary file 1 — Additional file 1. Timeline in relation to contextual factors. [file 12909_2021_3037_MOESM1_ESM.docx]
