# Supplementary material for: “More than just a medical student”: a mixed methods exploration of a structured volunteering programme for undergraduate medical students
Source: BMC Med Educ. 2022 Jan 3;22:1. doi: 10.1186/s12909-021-03037-4 (PMC8721190; doi:10.1186/s12909-021-03037-4)
Supplement: Supplementary file 2 — Additional file 2. Student and Supervisors, teaching fellows and clinical support surveys. [file 12909_2021_3037_MOESM2_ESM.docx]

## Additional File 2 – Student and Supervisors, teaching fellows and clinical support surveys

**Student Survey**

Thank you for your contribution to ICSM-Volunteering

We would like to explore your experiences so that we can learn from them and share lessons for the future. We would be grateful if you would complete the following questionnaire.

It should take approximately 15-20 minutes to complete. No identifying information is collected. Your responses will be analysed by researchers from Imperial College's Medical Education Research Unit (MERU) and any potentially identifying information removed prior to sharing with the ICSM-V Team.

Completing this questionnaire will be taken as consent for your answers to be used for evaluation and research purposes. You do not need to complete this questionnaire, and non-completion will not harm you in any way.

Please contact [k.leedham-green@imperial.ac.uk](mailto:k.leedham-green@imperial.ac.uk) if you have any questions.

What year are you in? (dropdown menu)

Where were you based? (dropdown menu)

Please tell us about your motivations for volunteering (freetext box)

Please describe your hopes and fears before your started (freetext box)

How did your actual experience compare with your expectations? (freetext box)

What was your role(s) once you settled in? (freetext box)

Approximately when did you start volunteering (dd/mm/yyyy)?

How many days per week did you volunteer for?

When did/will you stop volunteering? (dd/mm/yyyy)

Why did/will you stop volunteering? (Tick as many as apply)

- I became unwell
- I didn't feel I was helping
- I was no longer needed
- I needed to prioritise my studies
- I had other obligations e.g. family/work
- The opportunity closed / was of fixed length
- Other

If you would like to say anything else about your reasons for stopping, please do so here (freetext box):

Please let us know about your supervision:

|  |  |  | Please select | | |  |
| --- | --- | --- | --- | --- | --- | --- |
|  |  |  | Yes | No | Unsure |  |
| Did you have a named clinical supervisor? |  |  |  |  |  |  |
| Did you have a named teaching fellow coordinator? |  |  |  |  |  |  |
| Did you attend an induction? |  |  |  |  |  |  |

To what extent do you agree with the following statements about your induction (skip if no induction) (1-5 stars)

|  |  |  |  |  |
| --- | --- | --- | --- | --- |
| The induction appropriately prepared me for my role |  |  |  |  |
| The induction increased my confidence in my role as a volunteer |  |  |  |  |
| I had sufficient opportunity to discuss which role I would like to take as a volunteer |  |  |  |  |
| I had sufficient opportunity to discuss any questions or concerns |  |  |  |  |
| I had adequate practical guidance (bleep system/ clothes washing/ attendance etc) |  |  |  |  |
| I had adequate PPE training |  |  |  |  |

Please share any insights into your induction (what helped, what was missing), giving as much detail as possible. (freetext box)

To what extent do you agree with the following statements (1-5 stars)

|  |  |  |  |  |
| --- | --- | --- | --- | --- |
| I felt adequately supervised throughout my volunteering |  |  |  |  |
| I had regular meetings with my supervisor(s) |  |  |  |  |
| I had a good rapport with my supervisor(s) |  |  |  |  |
| I felt able to approach my supervisor(s) for support about my role |  |  |  |  |
| I felt able to approach my supervisor(s) for personal support |  |  |  |  |

Please share any insights into your supervision. Please give as much detail as possible, but without names. (freetext box)

How do you feel your contribution(s) were valued by the team(s) you worked with? (freetext box)

What did you learn as a result of this volunteering experience? This might be about medicine, about your profession and others, about patients and families, about teams, about yourself, about service provision, healthcare systems, skills, or about life in general. Please give as much detail as possible.

We are particularly interested in your insights as to why learning as a volunteer was different to learning as student. (freetext box)

How do you think the experience of volunteering will influence you in the future? (freetext box)

Were there any other impacts (positive or negative) on you as a result of this volunteering experience? This might be to your health, to your well-being, or to how you feel about yourself or your profession.  (freetext box)

We are interested in analysing narratives of your experiences:

|  | Yes | No |
| --- | --- | --- |
| Did you keep a reflective diary, vlog or journal? |  |  |
| Would you consider sharing this with the research team? |  |  |
| Did you do this as part of a year 2 CRI project? |  |  |

Only share your reflective diary, vlog or journal if you are comfortable doing so. MERU will remove all identifying content and/or decontexualize your diary prior to analysis. If we use any illustrative quotes these will be checked back with you so that you can see how they are used in context before publication. You can withdraw your consent at any time.

This [information sheet](https://imperialcollegelondon.box.com/s/urwel9ffgawkncspgoan1f7dcjzmsq5j) explains your rights and how we will protect your privacy. If you consent, please email [MERU@imperial.ac.uk](mailto:meru@imperial.ac.uk) with your completed [consent form](https://imperialcollegelondon.box.com/s/ik3ulikqmgl3hn28sgnmy9qxqvomsfpi), or email [k.leedham-green@imperial.ac.uk](mailto:k.leedham-green@imperial.ac.uk) if you have any questions. A member of MERU will then contact you with instructions on how to share your diary/vlog/journal.

We would also like to conduct some online focus groups. Please email [k.leedham-green@imperial.ac.uk](mailto:k.leedham-green@imperial.ac.uk) to express your interest, with "ICSM-V FOCUS GROUP" in the email title and the place(s) where you were volunteering in the email body.

| Overall, how would you rate your volunteering experience? |
| --- |
| \|  \| \| --- \| \|  \| |

Finally, we would like to ask some demographic questions so that we can check that this programme was experienced equitably. You may skip any of these questions.

What gender do you identify as? (dropdown menu)

Do you identify as black or minority ethnic? (yes/no/prefer not to say)

Do you identify as coming from a social background where few people go to university? (yes/no/prefer not to say)

Is there anything else you would like to say, including feedback on this survey? (yes/no/prefer not to say)

## Supervisors, teaching fellows and clinical support survey


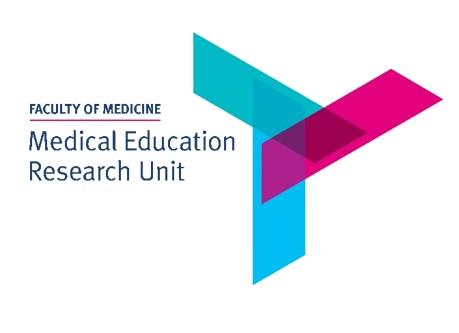


Thank you for your contribution supporting the ICSM-Volunteer Programme.

We would like to explore your experiences so that we can learn from them and share lessons for the future. We would be grateful if you would complete the following questionnaire.

It should take approximately 15-20 minutes to complete. Your responses will be analysed by researchers from Imperial College's Medical Education Research Unit and any potentially identifying information removed prior to sharing with the ICSM-V Team. Completing this questionnaire will be taken as consent for your answers to be used for evaluation and research purposes. You do not need to complete this questionnaire, and non-completion will not harm you in any way.

Please contact [k.leedham-green@imperial.ac.uk](mailto:k.leedham-green@imperial.ac.uk) if you have any questions.

Where are you based?

What is your job role?

- Nurse
- Doctor
- Undergraduate Teaching Coordinator
- Other managerial / administration
- Other

What was your role within the ICSM-Volunteering Programme? (select as many as apply)

- Local Programme Coordinator
- Teaching Fellow Coordinator
- Administrative Support
- Clinical Supervisor
- Other

If you were involved in leading/coordinating your local programme, please describe it (induction, teams, tasks, support/supervision etc). Alternatively, send documentation to [MERU@imperial.ac.uk](mailto:MERU@imperial.ac.uk)

Please describe your hopes and fears before the volunteers started (freetext box)

How did the reality of having ICSM volunteers compare to your expectations? (freetext box)

What role(s) did the medical students undertake? (that you supported/supervised) (freetext box)

What do you feel the student volunteers learned or gained? How did they progress? Please give as much detail as possible. (freetext box)

To what extent do you agree with the following statements (1-5 stars)

|  |  |  |  |  |
| --- | --- | --- | --- | --- |
| I felt supported by ICSM Volunteering Programme in my role with students |  |  |  |  |
| I had regular meetings with the student volunteer(s) |  |  |  |  |
| I developed a good rapport with the student volunteer(s) |  |  |  |  |
| I provided role support to student volunteer(s) |  |  |  |  |
| I provided personal support to student volunteer(s) |  |  |  |  |

Please share any insights into the supervision/support you provided. Please give as much detail as possible, but without names. (freetext box)

Please share any comments or insights into how the ICSM-V programme was organised. (freetext box)

What was most helpful about having student volunteer(s) at your site? (freetext box)

How could students be more helpful in the future if a second wave or other major incident happens? (freetext box)

Are there any aspects of this programme that you feel should be incorporated into the formal curriculum? How might this work? (freetext box)

Were there any other impacts (positive or negative) on you or your team as a result of the student volunteers? (freetext box)

| Overall, how would you rate the ICSM Volunteering Programme? |
| --- |
| \|  \| \| --- \| \|  \| |

Is there anything else you would like to say, including feedback about this survey? (freetext box)

If you have kept a reflective diary, vlog or journal that you are comfortable sharing, our researchers would be interested in learning from it. MERU will remove all identifying content and/or decontexualize your diary prior to analysis. If we use any illustrative quotes these will be checked back with you so that you can see how they are used in context before publication. You can withdraw your consent at any time.

This [information sheet](https://imperialcollegelondon.box.com/s/urwel9ffgawkncspgoan1f7dcjzmsq5j) explains your rights and how we will protect your privacy. If you consent, please email [MERU@imperial.ac.uk](mailto:meru@imperial.ac.uk) with your signed [consent form](https://imperialcollegelondon.box.com/s/ik3ulikqmgl3hn28sgnmy9qxqvomsfpi), or email [k.leedham-green@imperial.ac.uk](mailto:k.leedham-green@imperial.ac.uk) if you have any questions. A member of MERU will then contact you with instructions on how to share your diary/vlog/journal.
